# Identification of the Avian *RBP7* Gene as a New Adipose-Specific Gene and RBP7 Promoter-Driven GFP Expression in Adipose Tissue of Transgenic Quail

**DOI:** 10.1371/journal.pone.0124768

**Published:** 2015-04-13

**Authors:** Jinsoo Ahn, Sangsu Shin, Yeunsu Suh, Ju Yeon Park, Seongsoo Hwang, Kichoon Lee

**Affiliations:** 1 Department of Animal Sciences, The Ohio State University, Columbus, Ohio, United States of America; 2 Interdisciplinary Ph.D. Program in Nutrition, The Ohio State University, Columbus, Ohio, United States of America; 3 Life and Industry Convergence Research Institute, Pusan National University, Miryang, Gyeongnam, Republic of Korea; 4 Department of Biological Sciences, The Ohio State University, Columbus, Ohio, United States of America; 5 Animal Biotechnology Division, National Institute of Animal Science, Suwon, Gyeonggi, Republic of Korea; Cincinnati Children's Hospital Medical Center, UNITED STATES

## Abstract

The discovery of an increasing number of new adipose-specific genes has significantly contributed to our understanding of adipose tissue biology and the etiology of obesity and its related diseases. In the present study, comparison of gene expression profiles among various tissues was performed by analysis of chicken microarray data, leading to identification of *RBP7* as a novel adipose-specific gene in chicken. Adipose-specific expression of RBP7 in the avian species was further confirmed at the protein and mRNA levels. Examination of the transcription factor binding sites within the chicken RBP7 promoter by Matinspector software revealed potential binding sites for adipogenic transcription factors. This led to the hypothesis that the RBP7 promoter can be utilized to overexpress a transgene in adipose tissue in order to further investigate the function of a transgene in adipose tissue. Several lines of transgenic quail containing a *green fluorescent protein* (*GFP*) gene under the control of the RBP7 promoter were generated using lentivirus-mediated gene transfer. The GFP expression in transgenic quail was specific to adipose tissue and increased after adipocyte differentiation. This expression pattern was consistent with endogenous RBP7 expression, suggesting the RBP7 promoter is sufficient to overexpress a gene of interest in adipose tissue at later developmental stages. These findings will lead to the establishment of a novel RBP7 promoter cassette which can be utilized for overexpressing genes of interest in adipose tissue *in vivo* to study the function of genes in adipose tissue development and lipid metabolism.

## Introduction

Adipose tissue is a highly interactive organ that stores energy in the body to sustain energy balance. Identification of adipose-specific genes and use of their promoters that can drive expression of genes in adipose tissue of transgenic animal models are of particular interest in studying functions of genes in adipose growth and development *in vivo*. The results from the studies using transgenic animal models could provide *in vivo* evidence that ultimately leads to the potential application to ameliorate obesity and to improve production efficiency of food animals. However, it is difficult to adapt conventional promoters to modulate adipocyte gene expressions selectively *in vivo* for medical and agricultural purposes.

Retinol binding proteins (RBPs) are a family of transporters for fat-soluble retinol (preformed vitamin A), which has diverse functions in vision, reproduction, and animal growth [1]. Among them, retinol binding protein (RBP7) is one of the cellular retinol binding proteins that play a role in cellular metabolism of retinol [2]. In our microarray and real-time PCR data using chicken tissue samples, expression of the avian *RBP7* gene was predominant in adipose tissue, which is a major reservoir for retinol. Further promoter analysis has shown that the RBP7 promoter contains regulatory elements for adipose-specific expression. Thus, the RBP7 promoter has been proposed as a novel adipose tissue-specific promoter. However, to our knowledge, there have been no reports on any transgenic livestock animal and poultry with adipose-specific expression of a transgene, beside our recent transgenic quail studies [3].

In this study, *RBP7* was identified as an adipose-specific gene in the avian species and transgenic quail expressing an *enhanced green fluorescent protein* (*eGFP*) gene in adipose tissue under the control of RBP7 promoter was generated in order to selectively target the transgene (*eGFP*) expression in adipose tissue. In addition to its adipose tissue specificity, the regulation of the transgene during the development of adipocytes was investigated. It has been suggested that the RBP7 promoter may stimulate GFP expression very strongly in the neck and abdominal adipose tissue of quail. Moreover, GFP expression was significantly high in mature adipocytes, indicating a late-temporal control by the RBP7 promoter. The avian transgenesis used in this study to produce adipose-specific fluorescent transgenic quail can be utilized to drive spatiotemporally controlled overexpression of target transgenes to discover the function of unknown genes during adipose tissue development *in vivo*.

## Materials and Methods

### Animal use

All procedures for animal care and use were approved by The Ohio State University Institutional Animal Care and Use Committee (IACUC). Quail, turkeys and chickens were maintained at the Ohio State Poultry House, The Ohio State University, Columbus, OH. For tissue collection, birds were euthanized by CO_2_ inhalation.

### Total RNA isolation and microarray analysis

Total RNA was isolated from chicken tissues using Trizol (Life Technologies Inc., Grand Island, NY) according to the manufacturer’s instructions. RNA quality and quantity were assessed by gel electrophoresis and NanoDrop (NanoDrop Technologies, Wilmington, DE), respectively. RNA samples for each tissue or organ, including fat, muscle, heart, lung, liver, kidney, brain, and spleen were pooled from three chickens and combined as one sample per tissue or organ to decrease individual variability. The pooled RNA samples were frozen and sent out to The Ohio State University Microarray Core Facility. These RNA samples were reverse-transcribed into cDNA and labeled. The labeled cDNAs were hybridized to the Affymetrix GeneChip Chicken Genome array chip which covers 32,773 transcripts corresponding to 28,000 chicken genes. The raw data were gathered and converted by Dr. Lianbo Yu from the Center for Biostatistics at The Ohio State University. Background correction and quantile normalization of the data were then conducted. The microarray results were returned as a relative gene expression per sample. The microarray data saved in an Excel file were further processed for ranking genes that are abundantly expressed in adipose tissue by the procedures described in our previous report [4].

### Quantitative Real-Time PCR

To confirm adipose-specific expression of RBP7 that was most highly expressed in adipose tissue based on an analysis of our microarray data, relative mRNA expression levels of RBP7 in various chicken tissues (n = 4) were assessed by quantitative real-time PCR (qPCR). In addition, for the quantification of gene expression in fat cell fractions, subcutaneous adipose tissue was collected from chicken (n = 4) and quail (n = 3) and the fractionation of stromal-vascular cell (SV) and fat cell (FC) was performed as described previously [5]. Both various chicken tissues and SV and FC fractions were gathered for RNA isolation using Trizol (Life Technologies Inc.). After conducting RNA isolation, RNA concentration was checked by nanodrop and 1 μg of total RNA for each sample was used for reverse transcription (RT) in 20 μL reaction following the instruction from the manufacturer of Moloney murine leukemia virus (M-MLV) reverse transcriptase (Invitrogen, Carlsbad, CA). The conditions for RT were 65°C for 5 min, 37°C for 52 min, and 79°C for 15 min. For qPCR, 2 μL of cDNA out of the 20 μL RT reaction were used as templates with the reaction system including AmpliTaq Gold polymerase (Applied Biosystems, Carlsbad, CA), GeneAmp 10×PCR Buffer (containing 100 mM Tris-HCl, pH 8.3), and 500-mM KCl. SYBR green was used to detect the amplification of products. Duplicate reactions were performed (25μL volume) on an ABI 7300 Real-Time PCR Instrument (Applied Biosystems). The condition of qPCR reaction was 95°C for 10 min, 40 cycles of 94°C for 15s, 57°C for 40s and 72°C for 30s, with an additional 82°C extension for 33s. A single major peak on the dissociation curves provided by the qPCR software indicated successful amplification. Chicken *β-actin* was used as a reference gene for the normalization of expression levels of RBP7 and other chicken genes. For the quantification of mRNA expression in quail, *ribosomal protein S13* (*RPS13*) was selected as a gene for normalization control, because of its stability described in previous reports [6, 7]. Relative expression of target genes was calculated using the 2^-ΔΔ^C_T_ (C_T_: threshold cycle) method [8]. The primer sequences for the chicken DLK1, β-actin and RPS13 were described in our previous reports [9, 10]. Other sets of primers used in this study are as follows: avian FABP4 forward primer 5’- GGG CAC CTG GAA GCT CCT T-3’and reverse primer 5’-TCT CAT CAA ACT CTT CAC CCA GCT-3’; avian SCD1 forward primer 5’- GAA CAT CAA CCC ACG GGA GAA-3’ and reverse primer 5’-AGC CCC AGG AGG CAC ATG A-3’; chicken PPARγ forward primer 5’-ACA TAA AGT CCT TCC CGC TGA CC-3’ and reverse primer 5’-TCC AGT GCG TTG AAC TTC ACA GC-3’; avian RBP7 forward primer 5’-CCC ACA GTC TAG CAA TGC CTG T-3’ and reverse primer 5’-GTT TCC ATG TTG GAA CCA ATG CT-3’; eGFP forward primer 5’-GCA TGG ACG AGC TGT ACA AGT AA-3’ and reverse primer 5’-CAT AAA GAG ACA GCA ACC AGG ATT-3’.

### Antibody production and Western blot analysis

An avian RBP7 polyclonal antibody was raised against an immunogen including amino acids 73–83 residues of chicken RBP7 protein in the rabbit by a custom antibody service (AbClon, Seoul, Korea). Protein isolation and Western blot analysis were conducted following the procedures from our previous report [11]. In detail, after SDS-PAGE and transfer to polyvinylidene fluoride (PVDF) membrane, the membrane was incubated overnight at 4°C with the RBP7 custom primary antibody at 1:3000 dilution in 1x Tris-buffered saline containing 0.05% tween-20 (TBST) with 4% non-fat dry milk. After washing in 1x TBST, Western blots were incubated with Rabbit IgG HRP-conjugated secondary antibody (R&D systems Inc., Minneapolis, MN) for 1 h at room temperature. The membrane was washed with 1x TBST before the addition of Amersham ECL plus Western Blotting Detection Reagents (GE Healthcare Biosciences, Pittsburgh, PA), and the blots were then exposed to Hyperfilm (GE Healthcare Biosciences) to visualize target proteins.

### Prediction of transcription factor binding sites

The DNA sequence of 5 kb upstream region from the start codon of the chicken *RBP7* (*cRBP7*) gene was obtained from the chicken genome browser at the UCSC (University of California, Santa Cruz) Genome Bioinformatics site (http://genome.ucsc.edu). With the DNA sequence, the binding sites of adipose tissue-specific transcription factors were predicted by using the MatInspector program (Genomatix Software GmbH, Munich, Germany). Among the 5 kb sequence, the 3 kb sequence from the start codon was used for a promoter as it had many binding sites of adipose tissue-specific transcription factors including PPARγ and C/EBPs.

### Cloning of cRBP7 promoter followed by lentiviral vector and particle production

The 3 kb sequence in the 5’ upstream region of *cRBP7* gene was amplified by PCR with a primer set of cRBP7P3K-F1 with ClaI site (forward primer, 5’-CGG TTA TCG ATG CAA TCA AAA TGC CAC TGA A) and cRBP7P3K-R1 with PacI site (reverse primer, 5’-CGG TTT TAA TTA ATG CTA GAC TGT GGG AAG GAG TTA-3’), and cloned into pCR2.1-TOPO vector (Invitrogen). The pCR2.1 recombinant vector was then digested with two restriction enzymes, ClaI and PacI, producing the 3 kb promoter fragment. This 3 kb promoter replaced a RSV promoter of a previously constructed pLTReGW lentiviral vector containing eGFP [12] after removing the RSV promoter from the pLTReGW lentiviral vector with the same restriction enzymes, ClaI and PacI. The final vector designed to express *eGFP* gene specifically in adipose tissue being driven by the RBP7 promoter was named as pLT-RBP7p3k-eGFP. Lentiviral particles were produced by co-precipitation of calcium phosphate and pLT-RBP7p3k-eGFP vector. In brief, on the day before transfection, 293 FT cells were plated on 100 mm culture dishes in the complete medium, which consisted of Dulbecco’s Modified Eagle Medium (DMEM; Life Technologies Inc.) supplemented with 10% fetal bovine serum (FBS; Life Technologies Inc.), 1% penicillin/streptomycin (pen/strep; Life Technologies Inc.), 0.1 mM MEM non-essential amino acids (Life Technologies Inc.), and 1 mM MEM sodium pyruvate (Life Technologies Inc.). To prepare transfection solution, 9 μg of pLT-RBP7p3k-eGFP, 9 μg of ViraPower Packaging Mix (Life Technologies Inc.), and 87 μl of 2M calcium solution (Clontech Laboratories Inc., Mountain View, CA) were added to a final volume of 700 μl of Sterile H_2_O (Clontech Laboratories Inc.) and then 700 μl of 2× HEPES-Buffered Saline (HBS) (Clontech Laboratories Inc.) were added dropwise while vortexing slowly. The transfection solution was then incubated at room temperature for 5 min and subsequently added dropwise to the complete medium. After 10 h of transfection, the medium was replenished with 5 ml of fresh complete medium. The supernatant was collected after 48 h and filtered through 0.22 μm pore size filters. The titer of lentiviral supernatants was measured by a standard ELISA method using the Lenti-X p24 Rapid Titer Kit (Clontech Laboratories Inc.) after the non-concentrated viral supernatants were serially diluted, which resulted in 1–10 ×10^7^ IFU/ml of an end point titer (data not shown). The filtered supernatant was then pelleted by centrifugation at 25,000 rpm for 2 h with an ultracentrifuge (L7-65R, Beckman Coulter, Fullerton, CA), resuspended in Opti-MEM as a 100× concentrated lentiviral particle soup, and stored as 40 μl aliquots at ‒80°C until use.

### Lentivirus-mediated gene transfer into stage X embryos

Newly laid eggs from wild-type Japanese quail were collected and cleaned with 70% ethanol. Eggs were positioned with their sides facing upward for approximately 4 h at room temperature to move embryos to the side. A window of about 5 mm in diameter was then made on the side using fine tweezers and 2–3 μl of the 100× concentrated lentiviral particle soup was microinjected into the subgerminal cavity of stage X embryos with a fully formed area pellucida [13]. The window was then sealed twice with paraffin film, and the eggs were incubated with the pointy end down until hatching.

### Hatching and mating after maturation

Of 184 stage X embryos microinjected with lentiviral particles containing pLT-RBP7p3k-eGFP, 29 quail hatched (15.8%) after 17 days of incubation. Among them, 17 hatchings (9.2%) survived up to 7 weeks until they sexually matured. These mature quail were mated with counter-sex wild-type quail, except for two cases in which two generation 0 (G0) quail were mated to each other, to construct 15 G0 founder lines. As a result, 30 transgenic generation 1 (G1) quail were produced from seven G0 founder lines. The success rate of transgenesis was between 5.1% and 14.3% for the seven lines (Table 1).

**Table 1:** Production of transgenic quail using lentivirus-mediated gene transfer into stage X quail embryos.

G0 founder quail		G1 lines (J)	G2 (Offspring of G1, K)				
Male	Female		Eggs	Hatched	Transgenic	Transgenic quail ID	
G0-8836	WT	J2	167	14	2 (14.3%)	K1,K13
G0-9201	WT	J4	100	41	5 (12.2%)	K3,K8,K12,K29,K30
WT	G0-9203	J7	132	78	8 (10.3%)	K2,K9,K15,K16,K18,K19,K25,K26
WT	G0-8897	J10	88	59	3 (5.1%)	K6,K24,K27
WT	G0-9204	J11	74	53	5 (9.4%)	K7,K11,K17,K20,K28
WT	G0-9202	J12	92	42	4 (9.5%)	K4,K10,K14,K22
G0-9198	G0-9205	J14	83	42	3 (7.1%)	K5,K21,K23

### Detection of the transgene in quail by PCR

To confirm the integration of the transgene in G1 and generation 2 (G2) quail, genomic DNA was extracted from the blood of hatched quail and subjected to genotyping PCR. Two primer sets of forward primers (5’- AAT CGC AAA ACC AGC AAG AAA -3’ and 5’- TGA CTG TAA CTC CTT CCC ACA GTC -3’) and reverse primers (5’- TTC AGT GGC ATT TTG ATT GCA -3’and 5’- TGA ACT TGT GGC CGT TTA CGT -3’) for ~300 bp fragments were used to detect the transgene construct by PCR. The PCR was performed in a volume of 25 μl, containing 200 ng of genomic DNA, 10 mM dNTP, 2.5 μl of 10× reaction buffer, 0.4 μM of forward and reverse primers, and 0.4 U of Taq DNA polymerase by using a MJ Research PTC-200 thermal cycler (MJ Research Inc., South San Francisco, CA). Cycle parameters for this PCR consisted of 2 min at 95°C, followed by 35 cycles of denaturation at 94°C for 30 sec, annealing at 55°C for 40 sec, extension at 72°C for 1 min, and a final extension of 10 min at 72°C. For negative and positive controls, genomic DNA extracted from wild-type quail and 4 pg of pLT-RBP7p3k-eGFP plasmid DNA were used as templates, respectively.

### Analysis of GFP expression

A total of seven 6-wk-old transgenic G2 quail and two wild-type quail were euthanized by CO_2_ inhalation, and their organs were collected including neck fat, abdominal fat, muscle, heart, lung, liver, kidney, spleen, brain, intestine, abdominal skin, and wing skin. For organ imaging of eGFP, its expression in transgenic quail was examined using a long-wave UV lamp (Blak-Ray B 100 AP/R, UVP, Upland CA, USA, radiation range 315−400 nm, peak at 365 nm). For cell culture studies, after primary stromal-vascular cells were cultured and differentiated as described in our previous report [14], adipocytes and undifferentiated preadipocytes were visualized under a fluorescence microscope at post-differentiation day 3 and analyzed using AxioVision software (Carl Zeiss, Göttingen, Germany).

### Statistical analysis

Statistical analyses were conducted by using statistical software programs as follows: Comparison of two means was performed by Student’s t-test. One-way ANOVA built in SAS 9.4 software program (SAS Institute Inc.) was used to compare multiple means followed by a Tukey’s post hoc test. *P*-value was <0.05 for the minimum level of significance.

## Results

### RBP7 is a novel adipose-specific gene discovered in comparative microarray analysis

**Fig 1:**
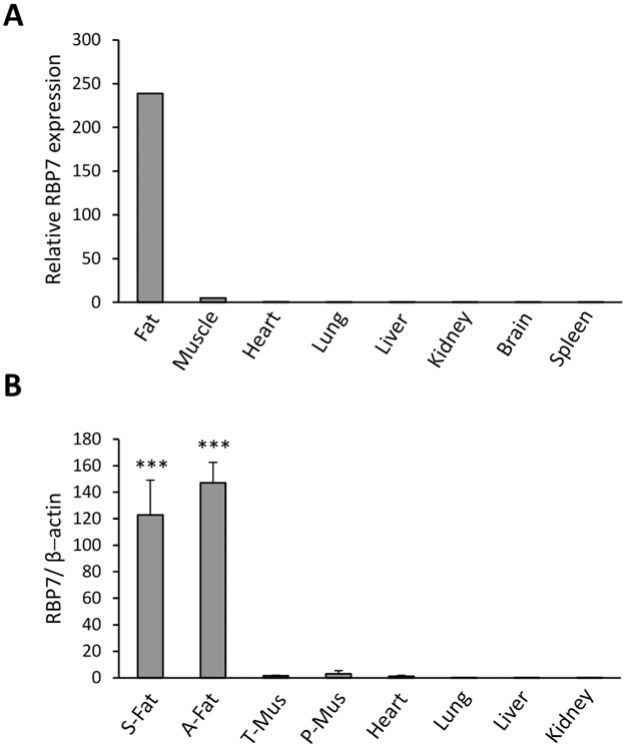
Expression of RBP7 mRNA in various chicken tissues. A) Relative RBP7 mRNA expression values from chicken cDNA microarray (n = 3, pooled from three chickens for each tissue). B) Measurement of RBP7 mRNA expression in chicken tissues by qPCR (n = 4) which is normalized to *β-actin* as a housekeeping gene. S-Fat: subcutaneous fat, A-Fat: abdominal fat, T-Mus: thigh muscle, P-Mus: pectoralis major muscle.

**Fig 2:**
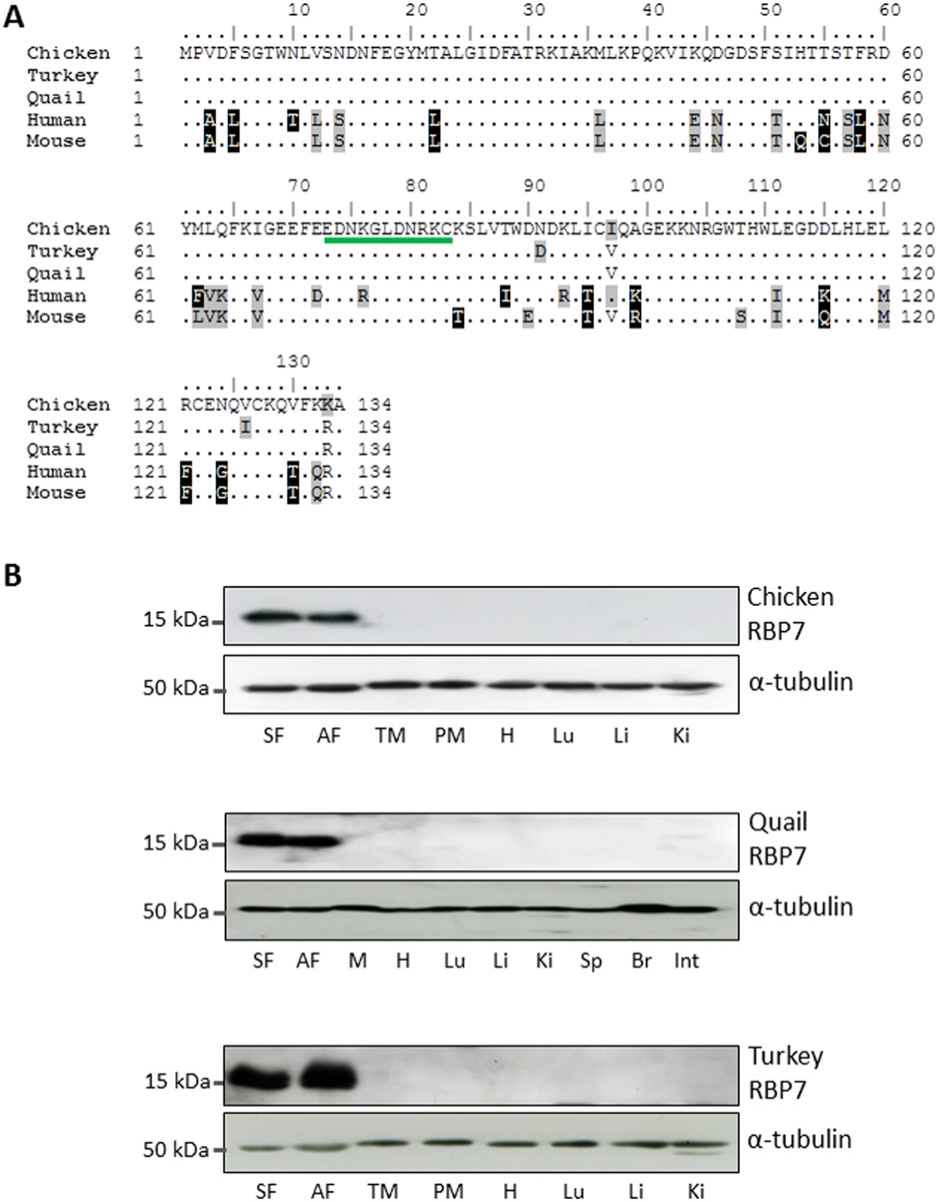
Comparative analysis of RBP7 sequence and protein expression in avian species. A) Comparison of RBP7 amino acid sequences of chicken (GenBank accession number XP_417606.4), turkey (XP_003212313.1), quail (corresponding to KP026122), human (NP443192.1) and mouse (NP071303.1). An epitope sequence detected by a custom RBP7 antibody is underlined from residue 73 to 83. B) Western blot analysis of RBP7 protein expression in chicken, quail and turkey tissues, respectively. *α-tubulin* was used as a loading control. SF: subcutaneous fat, AF: abdominal fat, TM: thigh muscle, PM: pectoralis major muscle, H: heart, Lu: lung, Li: liver, Ki: kidney, Sp: spleen, Br: brain, Int: intestine.

To identify novel adipose-specific genes, an analysis of chicken microarray was performed comparing expression levels of genes among various tissues. *RBP7* was identified as the first-ranked gene among 28,000 chicken genes (data not shown), by showing 238-fold higher expression in chicken adipose tissue compared to an average value in other chicken tissues (Fig 1A). This result was confirmed by qPCR, which showed 120-fold and 150-fold higher RBP7 expression in chicken subcutaneous and abdominal adipose tissue compared to an average level in other chicken tissues (Fig 1B). Western blot analysis further revealed an exclusive expression pattern of the chicken RBP7 (cRBP7) 15.57-kDa protein in neck (or subcutaneous) and abdominal adipose tissue, which is highly consistent in other avian species, quail and turkey (Fig 2B). Predicted amino acid sequence from quail RBP7 nucleotide sequence deposited to GenBank by this study (GenBank accession number KP026122) exhibited significant similarity to chicken and turkey sequences (S1 Fig), with only two amino acid differences in both cases, and an epitope sequence detected by our custom RBP7 antibody is conserved among avian species (Fig 2A). According to our comparative study of three avian species, *RBP7* was discovered as a novel adipose-specific gene whose mRNA and protein expression is specific to adipose tissue.

### A promoter region of RBP7 gene contains sequence elements for adipose-specific expression

**Fig 3:**
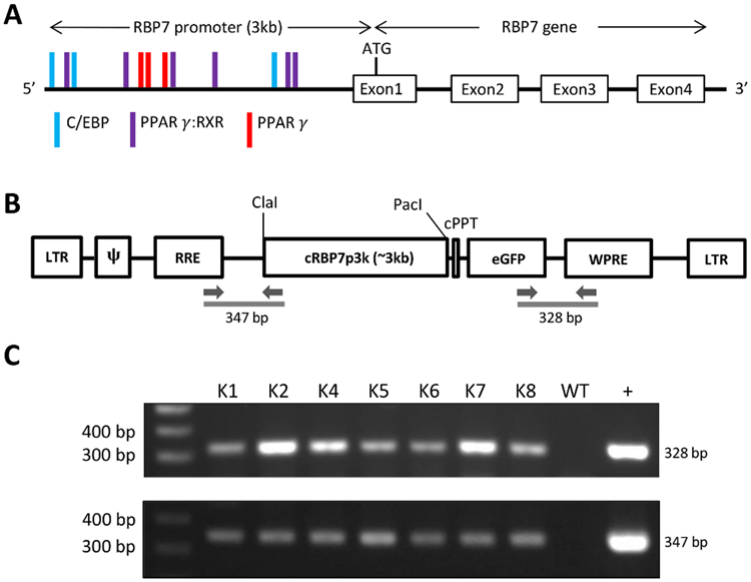
Analysis of RBP7 promoter, vector construct containing RBP7 promoter-eGFP, and PCR genotyping of transgenic quail. A) RBP7 promoter with regulatory elements interacting with major transcription factors in adipose tissue. B) Schematic representation of the pLT-RBP7p3k-eGFP vector. Restriction sites, ClaI and PacI are depicted. LTR, long terminal repeat; ψ, packaging signal; RRE, rev-response element; cPPT, central polypurine tract of HIV-1; eGFP, enhanced green fluorescent protein; WPRE, woodchuck hepatitis virus posttranscriptional regulatory element. The arrows represent the location of forward and reverse primers used for genotyping PCR. C) Genotyping PCR of transgenic and wild-type quail using the two sets of primers depicted in the figure of B). WT, wild-type quail; +: pLT-RBP7p3k-eGFP plasmid DNA (positive control).

In light of specificity of RBP*7* for adipose tissue, its promoter region was analyzed to find *cis*-acting elements that could be involved in adipose development. As predicted by the MatInspector program, the 3kb upstream promoter region from the start codon of the *cRBP7* gene was shown to have many *cis*-acting sequence elements that contain binding sites for PPARγ and C/EBP which are major transcription factors responsible for adipose-specific expression of multiple genes during adipose development (Fig 3A). It suggests that the endogenous cRBP7 promoter not only contains regulatory elements for the adipose-specific expression of *cRBP7* gene, but also can be used to express target transgenes such as *GFP* gene in an adipose-specific manner.

### Adipose-specific expression of GFP under the control of RBP7 promoter in transgenic quail lines

**Fig 4:**
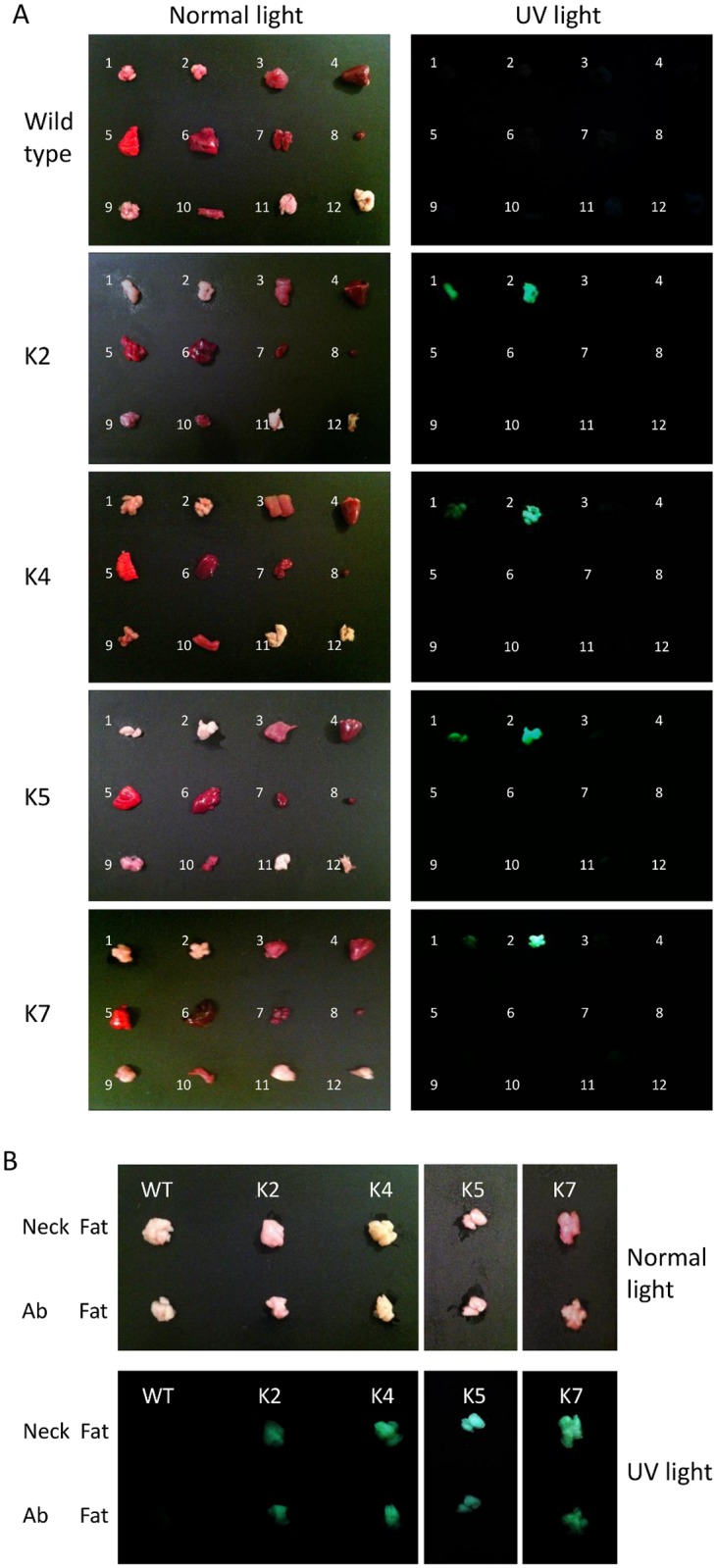
GFP expression in various tissues of transgenic quail. A) GFP expression in selected quail lines under normal and UV light. 1. Neck fat; 2. Abdominal fat; 3. Muscle; 4. Heart; 5. Lung; 6. Liver; 7. Kidney; 8. Spleen; 9. Brain; 10. Intestine; 11. Abdominal skin; and 12. Wing skin. B) Comparison of GFP expression in neck fat and abdominal fat tissues.

To prove adipose-specific regulation of RBP7 promoter *in vivo*, transgenic quail containing cRBP7 promoter–driven *GFP* transgene were generated. In particular, seven G1 transgenic lines were obtained as shown in genotyping PCR using primers designed to detect two transgene junctions, RRE-cRBP7 promoter and eGFP-WPRE portions of the lentiviral vector construct (Fig 3B and 3C). Among the seven transgenic lines, offspring in four lines (G2 transgenic quail) were born normally in a Mendelian ratio and showed strong GFP expression in neck and abdominal adipose tissue under the UV light with relatively even intensity across the different white adipose depots (Fig 4A and 4B), indicating adipose-specific expression of GFP under the control of RBP7 promoter.

### Expression of both endogenous RBP7 gene and GFP transgene increases during development of adipocytes in vivo and in vitro

**Fig 5:**
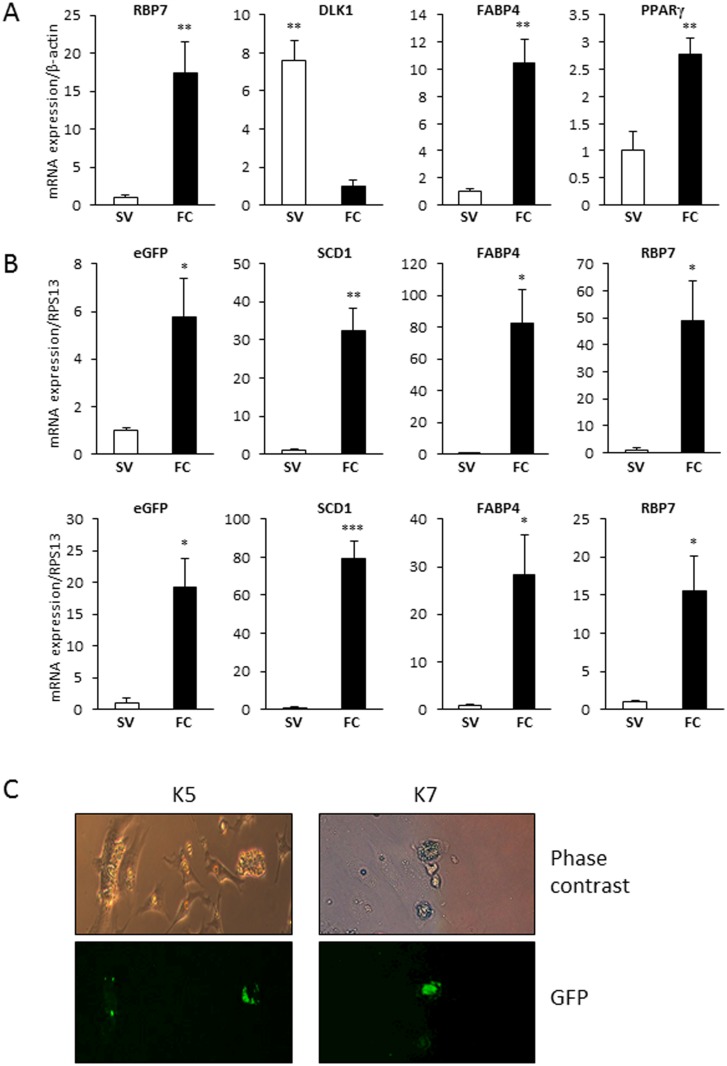
Quantitative analysis of gene expression in stromal-vascular (SV) and fat cell (FC) fractions and GFP images of cultured primary cells from transgenic quail. A) Quantification of RBP7, DLK1, FABP4, and PPARγ mRNA expression in SV and FC fractions from chicken adipose tissue by qPCR (n = 4). β-actin expression was used as a normalization control. B) qPCR analysis of SV and FC fractions from adipose tissue of selected transgenic G2 quail for mRNA expression of GFP, SCD1, FABP4, and RBP7 (n = 3). *Ribosomal protein S13* (*RPS13*) was selected as a reference gene. C) Phase contrast and GFP images of primary preadipocytes and adipocytes from K5 and K7 transgenic G2 quail.

To analyze RBP7 expression during avian adipogenesis *in vivo*, mRNA expression of chicken RBP7 in stromal-vascular (SV) cells containing mostly preadipocytes and fat cells was quantified by qPCR. Subcutaneous adipose tissue of the chicken was collected and fractionated into SV cells and fat cells. Successful fractionation was confirmed by examining marker gene expressions. As shown in Fig 4A, DLK1, a preadipocyte marker, was expressed 8 times higher in SV cells than in fat cells, and FABP4 and PPARγ, adipocyte markers were 10 and 3 times higher in fat cells than in SV cells, respectively, indicating effective fractionation. Chicken RBP7 mRNA expression was approximately 17-fold greater in fat cells compared to SV cells (Fig 5A), showing that cRBP7 is expressed higher in mature adipocytes rather than preadipocytes.

To compare expression patterns between the endogenous *RBP7* gene and the RBP7 promoter driven-*GFP* transgene during *in vivo* adipose development of transgenic quail, subcutaneous adipose tissue of two G2 transgenic quail lines were fractionated into SV cells and fat cells (Fig 5B). Adipocyte markers, FABP4 and SCD1 were highly expressed in fat cells compared to SV cells, indicating successful fractionation. RBP7 mRNA expression was significantly high in fat cells. Similarly, the target transgene (*GFP* gene) was also significantly highly expressed in fat cells.

In order to investigate the temporal expression of RBP7 and GFP during the development of adipocytes from transgenic quail *in vitro*, primary preadipocytes from the two selected transgenic quail lines were cultured and induced to be differentiated to adipocytes. The primary cell culture has shown that GFP expression is much more prominent in adipocytes than in neighboring preadipocytes (Fig 5C) indicating an adipocyte-specific GFP expression.

## Discussion

In general, most major tissues preferentially express approximately 100–500 tissue-specific genes [15]. Until recently, however, only a few genes have been identified as being adipose tissue-specific. Therefore, the discovery of new adipose-specific genes is essential to understand and advance adipose tissue biology. In the present study, we analyzed a chicken DNA microarray dataset followed by further confirmatory studies as described in our previously established method [4] to identify adipose-specific genes. This has led us to identify a novel adipose-specific *RBP7* gene and to hypothesize that the RBP7 promoter can be utilized to overexpress a transgene in adipose tissue for future investigation of the function of a transgene in adipose tissue development and lipid metabolism.

*Retinol binding protein 7* (*RBP7*) was identified in this study as a novel adipose-specific gene in the avian species and it is a member of the intracellular lipid-binding protein family. The *RBP7* gene has also been named cellular retinol binding protein IV (CRBP IV) in the human and cellular retinol-binding protein, type III (CRBP-III) in the mouse [16, 17]. Because dietary retinol (ROH) is a hydrophobic compound, its esterification to retinyl esters (RE) or combination with water-soluble retinol binding protein (RBP) is required for storage in the aqueous cytosol [18]. It has been shown that RBP1 plays a role in maintaining retinol storage in the liver and RBP2 is involved in retinoid metabolism in the intestine [16]. In addition, murine RBP7 has shown to be associated with retinol uptake and metabolism [18, 19] and 15–20% of total retinoid derivatives including retinols are stored in adipose tissue [20]. Based on these studies, it was assumed that the level of avian RBP7 may be high in adipose tissue where retinols are present with great abundance. Our results regarding adipose-specific expression of RBP7 support this assumption and RBP7 is a potential adipose-specific retinol-binding protein in the avian species. Furthermore, based on studies showing that retinoids are stored in adipocytes rather than in stromal-vascular fraction [21], higher expression of avian RBP7 in adipocytes than in stromal-vascular cells (Fig 5A) suggests the role of RBP7 on retinol metabolism in mature adipocytes rather than preadipocytes.

Considering adipose-specific expression of avian RBP7, it was hypothesized that the chicken RBP7 promoter may drive the expression of a transgene in an adipose-specific manner. Transgene expression targeting adipose tissue in transgenic mouse models have been achieved using the adipose protein 2 (aP2/FABP4) promoter [22–26]. However, it has been shown that the activity of the aP2 promoter can be found in other cell types, such as macrophages and cardiac myocytes, or other tissues including the heart, lung, and muscle [26]. In addition, an uneven distribution of expression of target genes across different fat pads has been reported [25]. Therefore, there has been an increasing demand for discovering new robust adipose-specific promoters. There have been attempts to generate an alternative adipose-specific promoter cassette such as a 4.9-kb adiponectin promoter cassette, using mouse models [27]. However, adiponectin gene expression was not as significant as RBP7 in chicken adipose tissue in our microarray analysis (data not shown), leading to an exclusion of adiponectin promoter cassette for the generation of transgenic avian species. In the current study, the exclusive expression of avian RBP7 protein in adipose tissue along with the presence of several binding sites for adipose-specific transcription factors, PPARγ and C/EBP in the promoter region of RBP7 suggests the RBP7 promoter will be a reliable promoter for driving overexpression of a transgene specifically in adipose tissue.

Quail has been developed as a model system for conducting genetic modifications due to its high egg-laying capacity, a short incubation period, and an earlier sexual maturation [3, 28]. Particularly, Japanese quail (*Coturnix c*. *japonica*) have been used to study the function of target genes in adipose tissue in light of the fact that *de novo* lipogenesis occurring primarily in the liver and secondarily in adipose tissue is conserved in both humans and avian species; whereas, it mainly takes place in adipose tissue in many other animals [3,29]. Also, there is no obvious difference in fatty acid synthesis and breakdown between humans and avian species [7, 14, 30]. Therefore, it is useful to generate a transgenic quail model to identify adipose-specific promoters that increase gene expression in adipose tissue and to study the development of adipose tissue in developing quail. In addition, lentiviral vectors have been used for gene therapy and adopted for the generation of transgenic quail, because of their efficient infection mechanisms without further manipulations [12, 31]. Obtaining advantages of lentivirus, transgenic quail could be generated to improve the efficiency of germline integration of *GFP* reporter gene under the regulation of RBP7 promoter. The expression of GFP was relatively even across different fat pads. Much weaker or no GFP images generated from tissues of some lines may be due to undesirable chromosome integration or an insertion of truncated transgene constructs into a chromosome (S2 Fig). In addition, during the development of adipose tissue, GFP expression was significantly high in mature adipocytes suggesting the regulatory role of RBP7 promoter in mature adipocytes.

In summary, the expression of *GFP* transgene under the regulation of RBP7 promoter follows the adipose-specific tissue distribution pattern of the expression of endogenous *RBP7* gene as well as its predominant expression pattern in adipocytes over preadipocytes within adipose tissue. These results suggest that the RBP7 promoter present in the transgene construct used for the generation of transgenic quail contains sufficient information to ensure developmental stage- and tissue-specific activity of the RBP7 promoter. Fundamental understandings gained from these findings will establish the construct of RBP7 promoter and a transgene as a new tool to overexpress transgenes specifically in adipose tissue *in vivo*, targeting adipocytes at later stages of development. It will lead to elucidating the function of a transgene in fat accretion in adipose tissue, thereby providing potential target genes for developing anti-obesity drugs and strategies and for selecting superior lines of livestock with less fat accretion and improved carcass quality.

## Supporting Information

S1 Fig Alignment of RBP7 nucleotide sequences. Nucleotide alignment using chicken (GenBank accession number XM_417606.4), turkey (XM_003212265.1), quail (KP026122), human (NM052960) and mouse (NM022020.2) sequences.
(TIF)

S2 Fig GFP expression in various tissues of non-transgenic quail. A) Selected quail lines without GFP expression. 1. Neck fat; 2. Abdominal fat; 3. Muscle; 4. Heart; 5. Lung; 6. Liver; 7. Kidney; 8. Spleen; 9. Brain; 10. Intestine; 11. Abdominal skin; and 12. Wing skin. B) Measurement of GFP expression in neck fat and abdominal fat tissues.
(TIF)
