# Supplementary figures and images for: Identification of the Avian RBP7 Gene as a New Adipose-Specific Gene and RBP7 Promoter-Driven GFP Expression in Adipose Tissue of Transgenic Quail
Source: PLoS One. 2015 Apr 13;10(4):e0124768. doi: 10.1371/journal.pone.0124768 (PMC4395105; doi:10.1371/journal.pone.0124768)

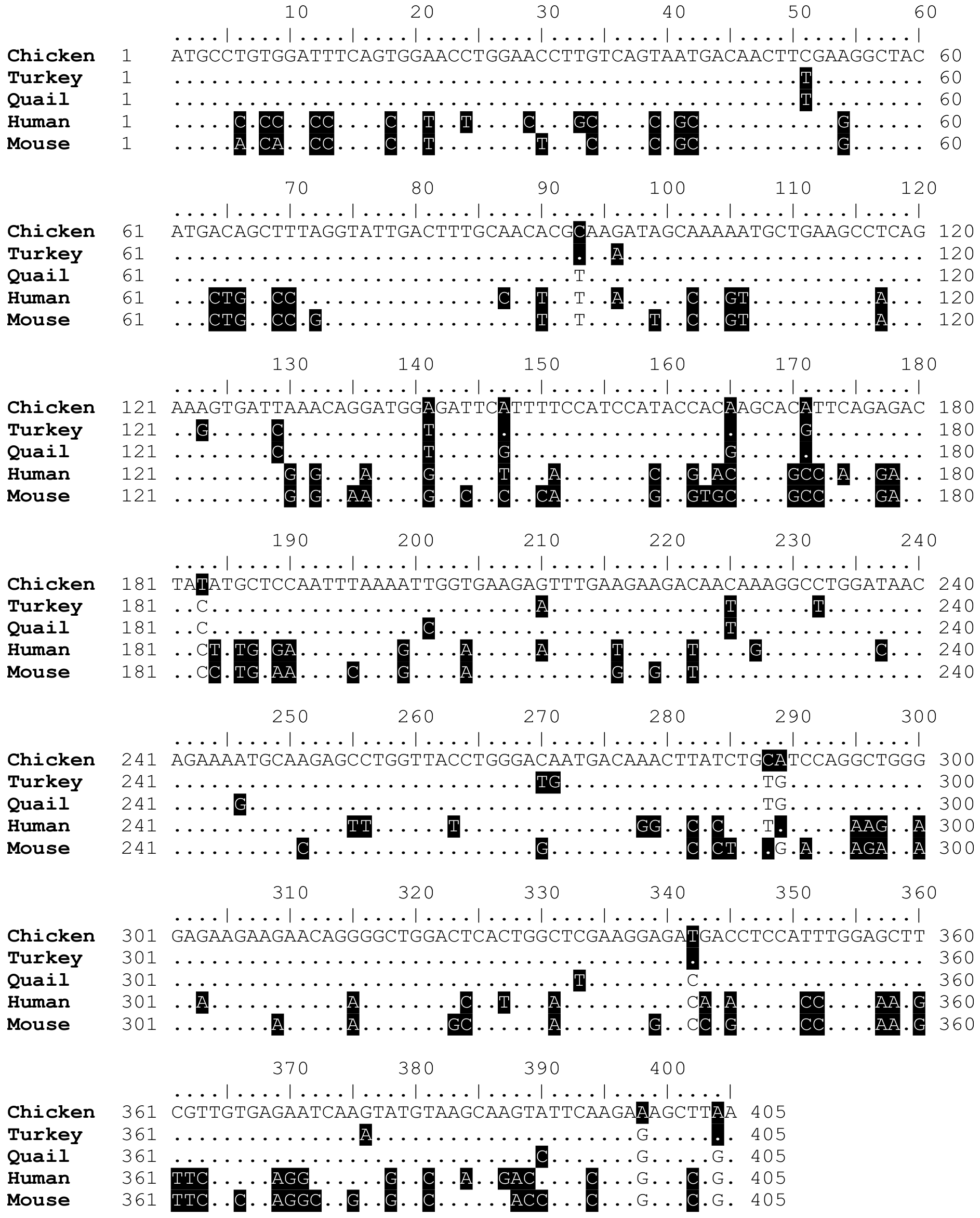

Supplement: S1 Fig — Nucleotide alignment using chicken (GenBank accession number XM_417606.4), turkey (XM_003212265.1), quail (KP026122), human (NM052960) and mouse (NM022020.2) sequences. (TIF) [file pone.0124768.s001.tif]

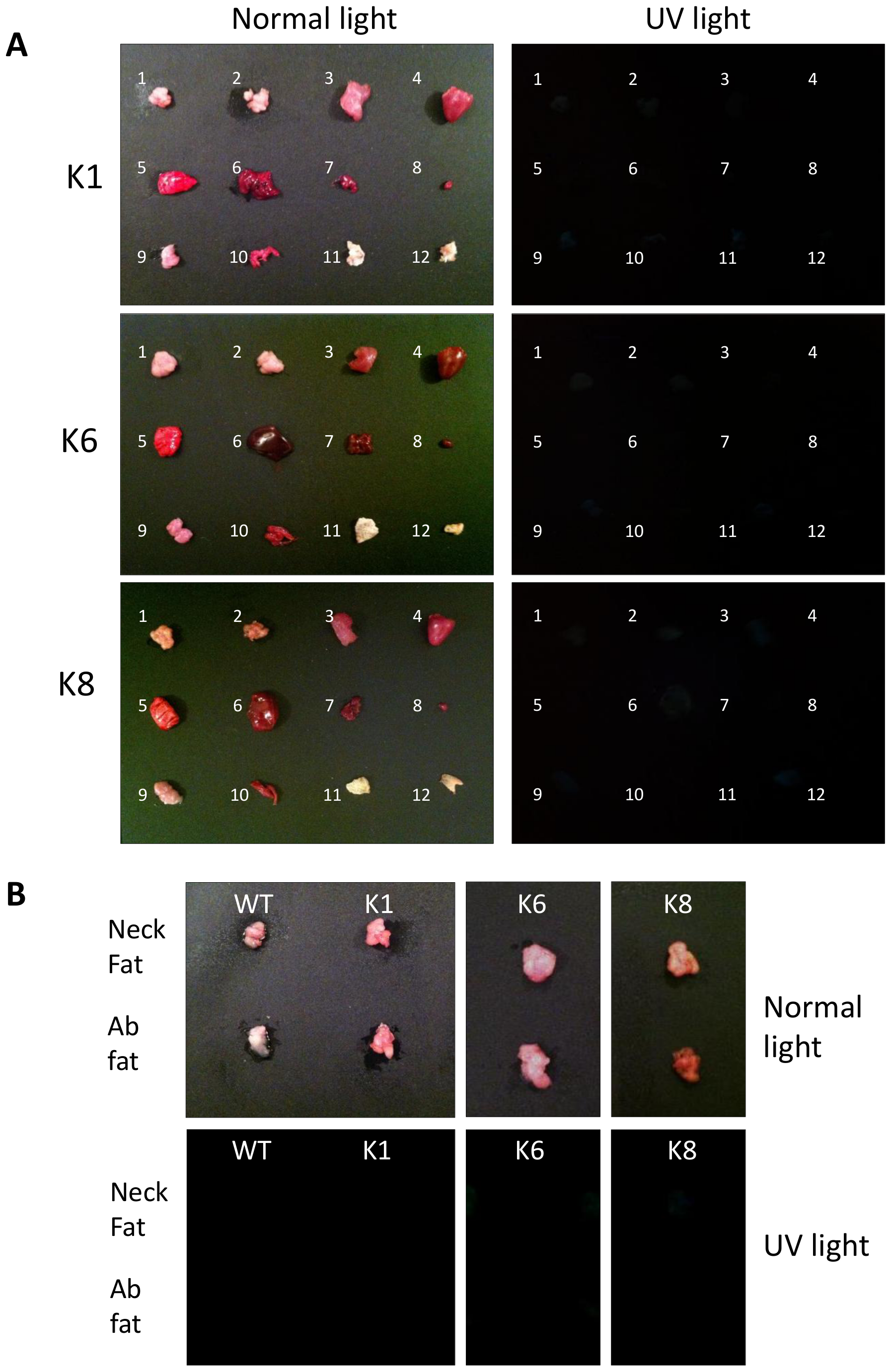

Supplement: S2 Fig — A) Selected quail lines without GFP expression. 1. Neck fat; 2. Abdominal fat; 3. Muscle; 4. Heart; 5. Lung; 6. Liver; 7. Kidney; 8. Spleen; 9. Brain; 10. Intestine; 11. Abdominal skin; and 12. Wing skin. B) Measurement of GFP expression in neck fat and abdominal fat tissues. (TIF) [file pone.0124768.s002.tif]
